# Le purpura de Schamberg

**DOI:** 10.11604/pamj.2015.20.144.3021

**Published:** 2015-02-17

**Authors:** Amal Akazane, Badreddine Hassam

**Affiliations:** 1 Service de Dermatologie Vénérologie CHU Ibn Sina, Rabat, Maroc; 2 Faculté de Médecine et de Pharmacie Med V Souissi, Rabat, Maroc

**Keywords:** Purpura de Schamberg, dermatite, hyperpigmentation, Schamberg purpura, dermatitis, hyperpigmentation

## Image en medicine

Le purpura de Schamberg appelée également purpura pigmentaire progressif est décrite pour la première fois en 1901 par J.F. Schamberg chez un garçon âgé de 15 ans. La maladie de Schamberg est insidieuse et chronique, débutant de façon asymptomatique sur les membres inférieurs sous forme de plaques ou de placards hyperpigmentés brun rouges. ; L'examen histopathologique montre un infiltrat mononuclé périvasculaire à la partie supérieure du derme avec extravasation de globules rouges et hémosidérine, mais pas de nécrose fibrinoide ; la maladie survient chez un sujet d'âge moyen avec une prédilection féminine, rare avant la puberté ,l'identification d'un facteur déclenchant est difficile, même si des facteurs étiologiques comme hypersensibilité médicamenteuse, dermatite de stase, dermatite de contact ont été décrits ; L'évolution est chronique et le traitement est en général symptomatique. Nous rapportons le cas d'une patiente de 52 ans ;hypertendue depuis 2 ans bien suivie , et ayant un terrain d'insuffisance veineuse des membres inferieurs évoluant depuis 5 ans. Qui consulte pour des placards hyperpigmentés brunâtres prurigineux des deux membres inferieurs dont le début insidieux remonte à un an ;l'examen clinique trouve des plaques hyperpigmentées rouges brunâtres non infiltrées confluentes à contours polycycliques par endroit intéressant les jambes et s'étendant aux cuisses , sur terrain d'insuffisance veineuse, le reste de l'examen clinique était normal. Ainsi une biopsie cutanée a montré un infiltrat inflammatoire mononuclé au niveau du derme avec dépôt d'hémosidérine suite à l'extravasation des globules rouges ,en faveur du purpura de Schamberg. Un traitement symptomatique a été prescrit à base d'antihistaminques et émollients vu la notion de prurit rapporté par la patiente, l'évolution a été marquée par deux à trois poussées par an entrecoupées de longue période de rémission, avec un recul de trois ans.

**Figure 1:**
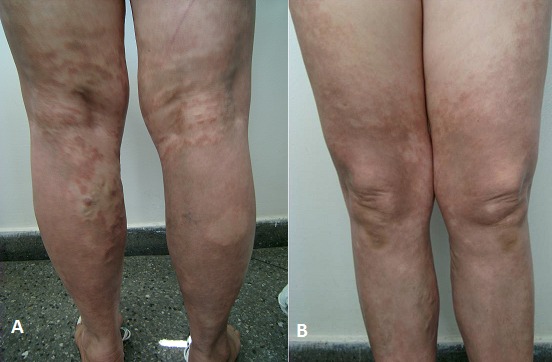
A) placards brunâtres des deux membres inférieurs; B) placards brunâtres s’étendant aux jambes

